# A multigene predictor of metastatic outcome in early stage hormone receptor-negative and triple-negative breast cancer

**DOI:** 10.1186/bcr2753

**Published:** 2010-10-14

**Authors:** Christina Yau, Laura Esserman, Dan H Moore, Fred Waldman, John Sninsky, Christopher C Benz

**Affiliations:** 1 Buck Institute for Age Research, 8001 Redwood Boulevard, Novato, CA 94945, USA; 2 Helen Diller Family Comprehensive Cancer Center, University of California, 2340 Sutter Street, San Francisco, CA 94143, USA; 3 Celera, LLC, 1401 Harbor Bay Parkway, Alameda, CA 94502, USA

## Abstract

**Introduction:**

Various multigene predictors of breast cancer clinical outcome have been commercialized, but proved to be prognostic only for hormone receptor (HR) subsets overexpressing estrogen or progesterone receptors. Hormone receptor negative (HRneg) breast cancers, particularly those lacking HER2/ErbB2 overexpression and known as triple-negative (Tneg) cases, are heterogeneous and generally aggressive breast cancer subsets in need of prognostic subclassification, since most early stage HRneg and Tneg breast cancer patients are cured with conservative treatment yet invariably receive aggressive adjuvant chemotherapy.

**Methods:**

An unbiased search for genes predictive of distant metastatic relapse was undertaken using a training cohort of 199 node-negative, adjuvant treatment naïve HRneg (including 154 Tneg) breast cancer cases curated from three public microarray datasets. Prognostic gene candidates were subsequently validated using a different cohort of 75 node-negative, adjuvant naïve HRneg cases curated from three additional datasets. The HRneg/Tneg gene signature was prognostically compared with eight other previously reported gene signatures, and evaluated for cancer network associations by two commercial pathway analysis programs.

**Results:**

A novel set of 14 prognostic gene candidates was identified as outcome predictors: CXCL13, CLIC5, RGS4, RPS28, RFX7, EXOC7, HAPLN1, ZNF3, SSX3, HRBL, PRRG3, ABO, PRTN3, MATN1. A composite HRneg/Tneg gene signature index proved more accurate than any individual candidate gene or other reported multigene predictors in identifying cases likely to remain free of metastatic relapse. Significant positive correlations between the HRneg/Tneg index and three independent immune-related signatures (STAT1, IFN, and IR) were observed, as were consistent negative associations between the three immune-related signatures and five other proliferation module-containing signatures (MS-14, ONCO-RS, GGI, CSR/wound and NKI-70). Network analysis identified 8 genes within the HRneg/Tneg signature as being functionally linked to immune/inflammatory chemokine regulation.

**Conclusions:**

A multigene HRneg/Tneg signature linked to immune/inflammatory cytokine regulation was identified from pooled expression microarray data and shown to be superior to other reported gene signatures in predicting the metastatic outcome of early stage and conservatively managed HRneg and Tneg breast cancer. Further validation of this prognostic signature may lead to new therapeutic insights and spare many newly diagnosed breast cancer patients the need for aggressive adjuvant chemotherapy.

## Introduction

Hormone receptor-negative (HRneg) breast cancer accounts for 30% to 40% of all newly diagnosed breast malignancies and is clinically subdivided into either human epidermal growth factor receptor 2 (HER2/ERBB2)-positive or triple-negative (Tneg) breast tumors, and about 60% of the latter consist of basal-like breast cancers [1-4]. When characterized by histology or protein-, RNA- or DNA-based assays, HRneg and Tneg breast cancers are consistently found to be aggressive and heterogeneous subgroups that defy prognostic substratification [5-9]. Tneg and basal-like breast cancers represent about 15% of all newly diagnosed breast cancers and preferentially arise in younger women, African-Americans, and BRCA1 mutation carriers. Given their reputation for more invasive and proliferative characteristics, even early-stage HRneg and Tneg breast primaries are invariably treated with adjuvant systemic therapy. Since Tneg breast tumors lack clinically validated prognostic or predictive biomarkers, their systemic therapy consists of empiric combinations of toxic chemotherapy.

The metastatic potential of HRneg and Tneg breast cancers, unlike hormone receptor-positive (HRpos) breast cancer, is usually manifest within 5 years of primary tumor diagnosis, with or without adjuvant chemotherapy intervention [10-12]. For example, despite both primary and systemic treatment, patients with Tneg breast cancer have a median time to metastatic recurrence of fewer than 3 years and are more than three times as likely to die from metastases within 5 years [12]. Despite this aggressive tumor behavior, nearly two thirds of newly diagnosed early-stage (T_1,2 _N_0,1_) Tneg patients conservatively managed without adjuvant chemotherapy remain disease-free 5 or more years after diagnosis, indicating that most do not require systemic therapy for curative intent and illustrating the clinical heterogeneity intrinsic to this otherwise-aggressive form of HRneg breast cancer [13]. Since more than 60% of incident breast cancers (including HRneg and Tneg cases) in the US are localized at the time of diagnosis and therefore are amenable to curative management without unnecessary systemic therapy [14], the failure of both traditional and modern high-throughput analytical methods to prognostically stratify HRneg and Tneg breast cancers for more personalized and conservative management points to a high-priority need for additional biomarker discovery [9].

Many multigene breast cancer classifiers and outcome predictors have been introduced to date, but none has become universally accepted, although several have been standardized and commercialized [8,9]. Given the diversity of genes in these signatures, it is surprising that they demonstrate nearly 80% classification concordance with routine pathology-based classifiers of breast cancer into HRpos, HRneg, HER2-positive, and Tneg subgroups [9]. Owing to the predominance of HRpos breast cancers and the many molecular differences distinguishing good-risk (luminal A) from poor-risk (luminal B) HRpos breast cancers, most of the well-described multigene predictors contain gene modules known to regulate or execute cell proliferation [9,15]. Thus, these signatures are most effective at assigning recurrence risk to early-stage HRpos breast cancer patients whose prognoses can be estimated using a simple Ki67 index [15] or more accurately assessed using a multigene predictor enriched for regulators of DNA and cell cycle function [16]. Large-scale meta-analyses across heterogeneous breast cancer datasets analyzed on different expression microarray platforms of multigene signatures like the 70-gene Mammaprint signature (NKI-70) [6], Celera 14-gene metastasis score (MS-14) [16], 76-gene Veridex signature (EMC-76) [17], core serum response (CSR/wound) signature [18], Oncotype/Genomic Health recurrence score (ONCO-RS) [19], p53 [20], and genomic grade index (GGI) [21] have shown that their prognostic values are comparable when evaluated against HRpos breast cancers (with or without adjuvant treatment). Moreover, despite the disparity in their gene composition, their proliferation modules appear to be the common driving force behind their overall prognostic value [22,23]. As the majority of HRneg breast cancers are highly proliferative, these various multigene predictors fail to show any value in discriminating prognosis within this HR subtype, supporting the widespread call for newer prognostic signatures not dependent on proliferation modules [22,23].

One meta-analysis observed that higher expression of an immune response (IR) gene module associated with STAT1 (signal transducer and activator of transcription 1) mRNA expression was significantly associated with better HRneg clinical outcome by univariate and multivariate analyses, prompting recent speculation that impaired host IR drives the development of HRneg metastatic events [23]. Earlier investigators showed that a novel interferon (IFN)-regulated breast cancer gene cluster, including the transcriptional regulator STAT1, was associated with somewhat better prognosis cases relative to other basal-like breast cancers [24]. Shortly thereafter, a team employing a novel pattern recognition and gene selection method and interrogating three public microarray datasets (based on different platforms) containing 186 adjuvant therapy-naïve, regionally involved HRneg breast cancers identified a 98-gene IR cluster and a 7-gene IR module capable of specifying up to 25% of HRneg breast cancers (including several HERpos but few medullary breast cancers) with significantly reduced risk of distant metastasis [25]. While the larger IR-98 gene cluster contained a number of IFN-related genes, including STAT1, the compact IR-7 module appeared functionally related to, but prognostically distinct from, the two previously reported IFN and STAT1 gene clusters [23,24]. More recently, this IR-7 predictor was refined by assigning different weights to the individual genes, yielding a composite IR score whose value increases with better HRneg prognosis [26]. While the prognostic value of this IR score was thought to be independent of tumor infiltration by lymphocytes [26], high levels of lymphocyte infiltration have been found to be associated with reduced risk of metastasis in Tneg/basal-like breast cancers [27]; thus, the prognostic contribution of host stromal and immune cell elements within the primary tumor remains an open question awaiting additional study. Meanwhile, the urgency to identify that vast majority of early-stage HRneg breast cancer patients not destined for metastatic relapse and to spare them unnecessary chemotherapy compelled a subsequent unbiased microarray search among node-negative HRneg and Tneg breast tumors for genes predictive of distant metastatic relapse.

The present study describes a novel set of 14 such prognostic gene candidates identified from a training cohort of 199 node-negative, adjuvant treatment-naïve HRneg (154 Tneg) cases curated from three public expression microarray datasets generated on the same microarray platform. Independent validation of the unweighted multigene HRneg/Tneg prognostic index was performed on a different cohort of 75 node-negative, adjuvant-naïve HRneg cases curated from three additional public datasets generated on two different microarray platforms. This novel HRneg/Tneg signature is able to better discriminate validation cases destined for metastatic relapse in comparison with eight other reported signatures. Interestingly, this HRneg/Tneg multigene index lacks any proliferation module and shows modest but significant correlations with the previously reported IR, IFN, and STAT1 module genes. Although the reported IR, IFN, or STAT1 module genes are not components of the HRneg/Tneg signature, one gene component of this index (CXCL13) correlates significantly with each of the 7 IR module genes, indicating surrogate representation of the IR-7 module within the 14-gene HRneg/Tneg index. In keeping with the immune ontology of both IR and IFN/STAT1 gene signatures, network analysis of the HRneg/Tneg signature reveals that half of the 14 index genes are functionally linked to immune/inflammatory cytokine regulation.

## Materials and methods

### Selection of HRneg and Tneg prognostic gene candidates

A set of 199 adjuvant-naïve, node-negative (N_0_), estrogen receptor-negative breast cancers annotated for distant metastasis-free survival (DMFS) was identified as HRneg training cases from three published microarray studies similarly analyzed on the Affymetrix (Santa Clara, CA, USA) U133A platform ([GEO:GSE2034] [17], [GEO:GSE5327] [28], and [GEO:GSE7390] [29]). Clinical parameters (grade and tumor size) available from each of these training data sources are summarized in Table S1 in Additional file 1. Tumor HER2 status was assigned based on mean-centered, log2-transformed ERBB2 transcript levels (probe set ID 216836_s_at) within each data source, yielding 154 Tneg training cases.

For candidate discovery, an initial subset of 135 HRneg cases from [GEO:GSE2034 and GSE5327] was analyzed by two different biostatistical approaches. In the first, prediction analysis of microarrays (PAM) was applied to log2-transformed discovery data subset by data source. Approximately 300 top discriminating probes were identified within each data source, and common probes with PAM scores bearing the same sign within both data sources were selected. Additional candidates were selected on the basis of a Monte Carlo cross-validation procedure. The discovery data subset was Z-transformed independently within data source and combined. A minimum variation filter was applied, yielding approximately 14,000 probes where at least 10% of cases showed greater than twofold variation from the mean. The filtered data were randomly subdivided into learning and test groups controlled for the number of metastatic cases. Univariate Cox analysis was performed, and prognostic significance was assessed as the *P *value computed from the Wald statistic averaged over 100 iterations. Probes with a *P *value of less than 0.01 and consistent correlation with DMFS (that is, Cox coefficient bearing the same sign) in more than 80% of all paired learning and test groups over the 100 iterations were selected. Univariate and multivariate Cox regression of DMFS on Z-transformed gene expression was performed on the discovery data for candidates with known official gene symbol annotation identified from both approaches, and probes with consistent correlation with DMFS in both univariate and multivariate settings were chosen for further assessment in the remaining 64 HRneg training cases from [GEO:GSE7930]. Expression data from these 64 cases were RMA-normalized and mean-centered in Bioconductor R, and univariate Cox regression of DMFS on gene expression was performed. Candidates with consistent correlation with DMFS in both subsets of the training cohort were selected as final HRneg prognostic gene candidates for further validation. Tneg-specific prognostic gene candidates were similarly selected from 154 Tneg training cases: 108 cases were from the initial discovery subset [GEO:GSE2034 and GSE5237], and 46 cases were from the additional training subset [GEO:GSE7930].

### Prognostic assessment of HRneg/Tneg genes within the training cohort

Mean-centered, log2-scaled data from the three independent studies comprising the 199 training cases were merged using distance-weighted discrimination (DWD) [30]. Prognostic performance of individual HRneg/Tneg prognostic markers was assessed using univariate and multivariate Cox analyses as well as Kaplan-Meier analysis of each marker dichotomized at its median expression level. Expression indices of the HRneg- and Tneg-specific markers as well as the combined set of HRneg/Tneg markers were computed for each patient as follows:

$$\frac{\sum_{i\in P}x_{i}-\sum_{j\in N}x_{j}}{n}$$

where *x *is the DWD-transformed expression, *n *is the number of genes within the signature, and *P *and *N *are the set of markers with positive and negative correlations with increased hazard, respectively. Tumors were dichotomized into high-versus-low index groups by their respective indices (that is, 199 HRneg cases by HRneg index and 154 Tneg cases by Tneg index) as well as the combined HRneg/Tneg index using median and upper third quartile values, which were discovered to yield near-optimal signature performance. Kaplan-Meier analysis was performed and significance was assessed by the log-rank statistic. Also, Cox regression of DMFS on group identity was performed to estimate the hazard ratio (HR) between patient groups with high-versus-low signature index. Candidates were also prioritized by stepwise variable analysis. Briefly, candidates were added one at a time to the signature beginning with the gene most strongly correlated with DMFS by univariate Cox analysis (largest coefficient or minimum *P*). With each step, expression indices were computed for all possible additions and scored by univariate Cox regression to determine the optimal order of addition and candidate subset (largest coefficient or minimum *P*). Likewise, candidates were subtracted one at a time from the combined 14-gene HRneg/Tneg signature for prioritization comparison.

### Prognostic assessment of HRneg/Tneg gene signature within the validation cohort

The independent validation cohort consisted of 75 untreated, node-negative HRneg primary breast cancers annotated for DMFS pooled from three independent datasets ([GEO:GSE6532] [31], [EBI:E-TABM-158] [7], and NKI-295 [32]). Clinical parameters (grade and tumor size) available from each of these validation data sources are summarized in Table S1 in Additional file 1. Of these cases, 38 were analyzed on the Affymetrix platform ([GEO:GSE6532], [EBI:E-TABM-158]) whereas the remaining 37 were assayed on the Agilent Technologies, Inc. (Santa Clara, CA, USA) HU25K platform (NKI-295). Data generated on the Affymetrix platform were normalized using RMA and mean-centered independently within each data source, and Agilent data were converted to log2-scale and mean-centered. Chip annotation files were obtained from the Broad Institute (Cambridge, MA, USA) website [33], and within each data source, expression data were collapsed by gene symbols such that the expression of genes represented by multiple probes was computed as the average across probes. Of the 14 gene candidates identified from the Affymetrix platform-based training cohort, only one (PRRG3) could not be identified on the Agilent array platform. Processed expression data were mapped across platforms using gene symbols prior to combination using DWD. HRneg/Tneg candidates were mapped to the combined validation dataset by gene symbol, and prognostic performance of the HRneg/Tneg signature as an index was assessed by Kaplan-Meier and Cox regression analyses of the validation cohort dichotomized at the upper third quartile cut-point, which was once again found to give near-optimal signature performance.

### HRneg/Tneg signature comparisons with other multigene predictors

The HRneg/Tneg candidates were assessed in relation to eight other signatures: NKI-70 [6], MS-14 [16], CSR/wound-response [18], ONCO-RS [19], GGI [21,31], IR-7 [25,26], STAT1 cluster [23], and IFN cluster [24] (Table S2 in Additional file 2). Gene signatures were mapped to the training and validation datasets using gene symbols, and for each signature, an expression index was computed for each patient as follows:

$$\frac{\sum_{i\in P}x_{i}-\sum_{j\in N}x_{j}}{n}$$

where *x *is the DWD-transformed expression, *n *is the number of genes (within the signature) that are mapped to the dataset, and *P *and *N *are the set of markers with previously reported positive and negative correlations with increased hazard, respectively. Prognostic comparison of the signatures was performed in the validation cohort to avoid training bias toward the HRneg/Tneg candidates. For each signature, tumors were dichotomized into high-versus-low index groups by median values. Here, to ensure fair comparisons and minimize bias toward the newly identified candidates, the upper third quartile cut-point, which yielded near-optimal HRneg/Tneg signature performance, was not employed. Kaplan-Meier analyses were performed and significance was assessed by log-rank statistic. Cox regression of DMFS on group identity was used to estimate the HR between patient groups with high-versus-low signature values. Pearson correlations between the signature indices were performed using both training and validation cohorts. In addition, T cell- and B cell-specific gene signatures derived from human peripheral blood (Table S2 in Additional file 2) were employed to estimate the degree of leukocyte infiltration within the training and validation tumors [34]. Signatures were mapped to the training and validation datasets by gene symbol, and the average expression of each signature was computed. Pearson correlations between the T-cell and B-cell signatures and the HRneg/Tneg index and IR-7 signature were performed on both training and validation cohorts. To confirm these associations, the analyses were repeated using a more restricted set of lymphocyte genes consisting of classical T cell- and B cell-specific surface markers and co-receptors (highlighted in red/yellow in Table S2 in Additional file 2).

### Pathway analysis of HRneg/Tneg signature genes

Pathway Studio (Ariadne Inc., Rockville, MD, USA) was used to identify potential common upstream regulators and downstream effectors of the Tneg/HRneg candidates. Also, Ingenuity Systems (Redwood City, CA, USA) was employed to explore potential connections between candidates through the shortest path (at most, one additional node) of direct interactions.

## Results

### Training cohort selection and assessment of HRneg/Tneg prognostic candidates

Following the multistep protocol described in Materials and methods, 11 probes, representing 11 unique genes (CLIC5, CXCL13, MATN1, RPS28/ANKRD47, ABO, EXOC7, HAPLN1, PRRG3, PRTN3, RFXDC2, and RGS4), were identified as HRneg prognostic candidates from the training cohort of 199 HRneg cases. Likewise, 7 probes, representing 7 unique genes (CLIC5, CXCL13, MATN1, RPS28/ANKRD47, HRBL, SSX3, and ZNF3), were identified as Tneg prognostic candidates from the subset of 154 Tneg cases within the training cohort. Altogether, a non-redundant set of 14 genes demonstrating prognostic value in either the full HRneg training data or the Tneg subset was identified as HRneg/Tneg prognostic candidates (Table 1). Each of these 14 HRneg/Tneg genes showed prognostic significance by univariate Cox analysis in the pooled training cohort, but only half retained prognostic significance by multivariate analysis (Table 1). Interestingly, all but 2 (HAPLN1 and RGS4) of the 14 genes yielded negative Cox coefficients, indicating that for the majority of the HRneg/Tneg genes, higher transcript expression is associated with better prognosis (Table 1). Kaplan-Meier analysis revealed that, except for 3 genes (RPS28/ANKRD47, MATN1, and HAPLN1), all of the HRneg and Tneg candidates were able to dichotomize the training cohort into prognostic groups showing significant differences in DMFS (Figures S1 and S2 in Additional files 3 and 4).

**Table 1:** Prognostic performance of individual HRneg/Tneg gene candidates in the HRneg training cohort (*n *= 199)

Affymetrix ID	Gene symbol	Gene title	Univariate Cox analysis		Multivariate Cox analysis	
				
			Coefficient	*P *value	Coefficient	*P *value
204338_s_at	*RGS4*	Regulator of G-protein signaling 4	0.24	2.64 × 10^-3^	0.16	0.12
205242_at	*CXCL13*	Chemokine (C-X-C motif) ligand 13 (B-cell chemoattractant)	-0.19	1.90 × 10^-5^	-0.16	6.0 × 10^-4^
205523_at	*HAPLN1*	Hyaluronan and proteoglycan link protein 1	0.17	1.04 × 10^-3^	0.18	1.4 × 10^-3^
206821_x_at	*HRBL*	HIV-1 Rev binding protein-like	-0.48	6.96 × 10^-4^	-0.22	0.18
206904_at	*MATN1*	Matrilin 1, cartilage matrix protein	-0.50	8.97 × 10^-5^	-0.11	0.45
207341_at	*PRTN3*	Proteinase 3 (serine proteinase, neutrophil, Wegener granulomatosis autoantigen)	-0.41	2.64 × 10^-4^	-0.12	0.37
207666_x_at	*SSX3*	Synovial sarcoma, X breakpoint 3	-0.33	2.17 × 10^-3^	-0.42	5.8 × 10^-4^
208902_s_at	*RPS28///ANKRD47*	Ribosomal protein S28///Ankyrin repeat domain 47	-0.59	1.08 × 10^-3^	-0.56	4.7 × 10^-3^
212035_s_at	*EXOC7*	Exocyst complex component 7	-0.58	2.47 × 10^-4^	-0.42	2.4 × 10^-2^
216929_x_at	*ABO*	ABO blood group (transferase A, alpha 1-3-N-acetylgalactosaminyltransferase; transferase B, alpha 1-3-galactosyltransferase)	-0.44	3.26 × 10^-4^	-0.23	9.2 × 10^-2^
217628_at	*CLIC5*	Chloride intracellular channel 5///similar to chloride intracellular channel 5	-0.48	1.89 × 10^-4^	-0.24	0.13
218430_s_at	*RFXDC2*	Regulatory factor X domain containing 2	-0.47	2.57 × 10^-4^	-0.40	4.6 × 10^-3^
219605_at	*ZNF3*	Zinc finger protein 3	-0.34	4.85 × 10^-3^	-0.30	4.5 × 10^-2^
220433_at	*PRRG3*	Proline rich Gla (G-carboxyglutamic acid) 3 (transmembrane)	-0.47	4.95 × 10^-4^	-0.27	6.2 × 10^-2^

HRneg, hormone receptor-negative; Tneg, triple-negative.

To assess the prognostic value of these HRneg/Tneg genes taken together as a multigene signature, an index value was computed as the sign-corrected average expression of the individual candidates such that higher expression of the signature index would be expected to correlate with worse prognosis. Kaplan-Meier analysis revealed that index values computed from the 11 genes identified from the HRneg training cohort (HRneg index) or from the 7 genes identified from the Tneg subset (Tneg index) were able to dichotomize their corresponding training cohorts into significantly different DMFS outcomes using a median value cut-point (log-rank *P *= 2.04 × 10^-7 ^and 1.73 × 10^-5^, respectively). The HRneg/Tneg index, comprising the non-redundant set of all 14 HRneg and Tneg prognostic candidates, achieved an even more significant curve separation (log-rank *P *= 6.14 × 10^-8 ^in full training data and 1.63 × 10^-6 ^in Tneg subset). Cox regression confirmed that the hazard associated with the high 14-gene HRneg/Tneg index value (HR 4.23, 95% confidence interval [CI] 2.4 to 7.45; *P *= 6.2 × 10^-7 ^in full training data and HR 4.18, 95% CI 2.22 to 7.88; *P *= 9.7 × 10^-6 ^in Tneg subset) was greater than that associated with either the HRneg or the Tneg indices in their corresponding training cohorts (HR 3.93, 95% CI 2.25 to 6.86; *P *= 1.4 × 10^-6 ^and HR 3.56, 95% CI 1.92 to 6.61; *P *= 5.6 × 10^-5^, respectively).

**Figure 1:**
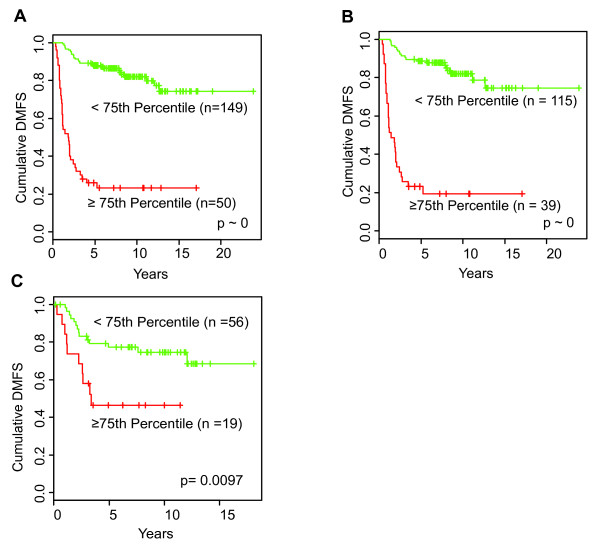
**Prognostic performance of the combined 14-gene HRneg/Tneg index in training and validation cohorts**. Kaplan-Meier plots of distant metastatic events dichotomized at the upper third quartile by high (red) or low (green) scores of **(a) **combined 14-gene HRneg/Tneg index in the full HRneg training cohort (*n *= 199), **(b) **combined 14-gene HRneg/Tneg index in the Tneg subset of the training cohort (*n *= 154), and **(c) **combined 14-gene HRneg/Tneg index in the full HRneg validation cohort (*n *= 75). Significant differences in survival between groups were determined by log-rank analysis. DMFS, distant metastasis-free survival; HRneg, hormone receptor-negative; Tneg, triple-negative.

Near-optimal curve separation was achieved using an upper third quartile (≥ 75th percentile) value as an HRneg/Tneg index cut-point (Figure 1). The Kaplan-Meier curves in Figure 1a and 1b show the full HRneg training cohort and its Tneg subset dichotomized at this third quartile cut-point into groups with significantly different DMFS outcomes based on the combined 14-gene HRneg/Tneg signature index. The Cox proportional HRs between high and low index groups were 9.13 (95% CI 5.5 to 15.2; *P *~0) in the full training data and 11 (95% CI 6.11 to 19.6; *P *~0) for the Tneg subset. As was observed using a median value cut-point, the prognostic performance of the combined 14 gene HRneg/Tneg index using a third quartile cut-point was superior or comparable to that of the individual HRneg or Tneg indices in their respective training cohorts (Figure S3 in Additional file 5).

Stepwise addition and subtraction analysis within the 199 training cohort prioritized the individual HRneg/Tneg genes comprising the 14-gene signature and revealed that 4 genes (CLIC5, EXOC7, RFXDC2, and SSX3) were consistently identified as the most significant contributors to the signature's prognostic value. Despite its prognostic significance in the multivariate Cox analysis, HAPLN1 was identified in the stepwise analysis as not providing additional prognostic value to the full 14-gene HRneg/Tneg signature.

### Validation cohort assessment of the HRneg/Tneg prognostic signature

An upper third quartile cut-point for the combined HRneg/Tneg signature index also proved near optimal in discriminating DMFS outcome within the 75-case validation cohort in which gene expression data were generated from two different microarray platforms. Figure 1c shows the Kaplan-Meier curves of the validation cohort dichotomized this way by the combined 14-gene HRneg/Tneg index. The Cox proportional HR between the high-versus-low HRneg/Tneg index groups in this validation cohort was 2.85 (95% CI 1.24 to 6.52; *P *= 0.013).

### Comparison of HRneg/Tneg signature with other multigene predictors

**Figure 2:**
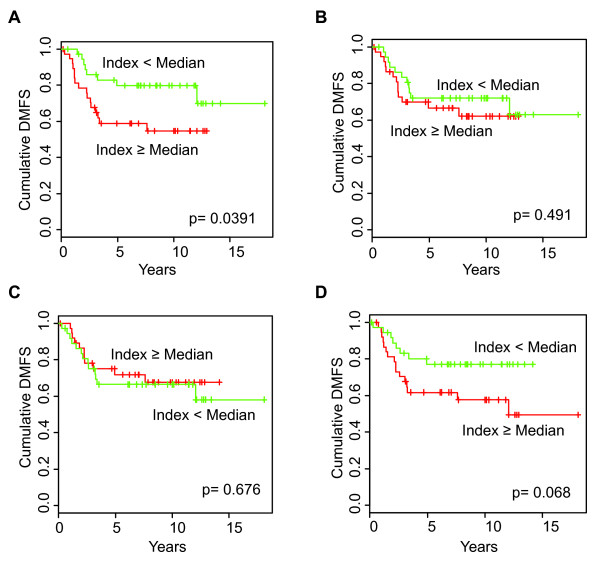
**Comparative prognostic performance of four different breast cancer gene signatures in the same validation cohort**. Kaplan-Meier plot of distant metastatic events among the validation cohort of 75 HRneg cases pooled from three different sources (Materials and methods) and dichotomized at the median signature value for high (red) or low (green) expression of the **(a) **HRneg/Tneg, **(b) **NKI-70, **(c) **MS-14, and **(d) **IR-7 indices. Significance of the difference in survival between groups was determined by log-rank analysis. DMFS, distant metastasis-free survival; HRneg, hormone receptor-negative; IR-7, immune response signature with 7-gene immune response module; MS-14, Celera 14-gene metastasis score; NKI-70, 70-gene Mammaprint signature; Tneg, triple-negative.

To compare the prognostic value of different signatures within the same population, a median cut-point value was used for each signature to dichotomize the validation cohort. Kaplan-Meier comparisons revealed that, of the nine signatures tested, only the HRneg/Tneg signature was able to significantly discriminate DMFS outcome (Figure 2a). Proliferation module-containing signatures like the NKI-70 (Figure 2b) and MS-14 (Figure 2c), known to be predictors of HRpos outcome [23,24], did not produce any prognostic separation in this HRneg population. The previously reported IR module, IR-7, though developed as an HRneg outcome predictor, trended toward discriminating DMFS outcome in this HRneg population only (Figure 2d). The log-rank *P *values of the Kaplan-Meier analyses and the Cox proportional HRs between the high-versus-low index groups for all nine multigene predictors in this validation cohort are shown in Table 2.

**Table 2:** Comparative prognostic performance of nine different breast cancer gene signatures in the HRneg validation cohort (*n *= 75)

Breast cancer gene signatures	Univariate Cox regression		Kaplan-Meier analysis
		
	Hazard ratio (95% confidence interval)	*P *value	Log-rank *P *value
HRneg/Tneg	2.38 (1.02-5.58)	0.045	0.039
STAT1 cluster [23]	2.06 (0.88-4.82)	0.095	0.088
IR-7 [25,26]	2.17 (0.93-5.07)	0.075	0.068
IFN cluster [24]	1.62 (0.71-3.71)	0.25	0.25
ONCO-RS [19]	1.45 (0.64-3.27)	0.37	0.36
GGI [21,31]	0.68 (0.30-1.53)	0.35	0.35
MS-14 [16]	0.84 (0.38-1.88)	0.68	0.68
NKI-70 [6]	1.33 (0.59-2.97)	0.49	0.49
CSR/wound [18]	0.68 (0.30-1.54)	0.36	0.35

CSR, core serum response; GGI, genomic grade index; HRneg, hormone receptor-negative; IFN, interferon; IR-7, immune response signature with 7-gene immune response module; MS-14, Celera 14-gene metastasis score; NKI-70, 70-gene Mammaprint signature; ONCO-RS, Oncotype/Genomic Health recurrence score; STAT1, signal transducer and activator of transcription 1; Tneg, triple-negative.

**Figure 3:**
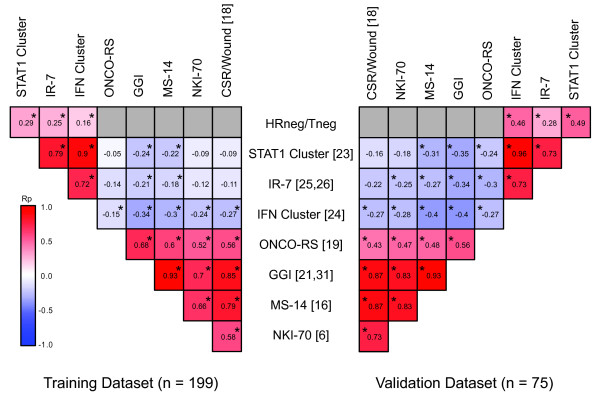
**Heatmap display of correlations between HRneg breast cancer expression of eight different gene signatures**. Each square within a pyramid displays the Pearson correlation coefficient (R_p_) between a pair of signatures as indicated on the horizontal and vertical axes. *Significant correlations (*P *< 0.05). Correlations computed from training cohort (*n *= 199) are displayed on the left, and correlations computed from validation cohort (*n *= 75) are displayed on the right. Red-blue color scale is used to reflect the magnitude of R_p_, with red denoting consistent positive and blue denoting consistent negative R_p _values across each cohort. Squares are colored grey (without R_p _values) for inconsistent associations (opposite R_p _directions) between cohorts. CSR, core serum response; HRneg, hormone receptor-negative; GGI, genomic grade index; IFN, interferon; IR-7, immune response signature with 7-gene immune response module; MS-14, Celera 14-gene metastasis score; NKI-70, 70-gene Mammaprint signature; ONCO-RS, Oncotype/Genomic Health recurrence score; R_p_, Pearson correlation coefficient; STAT1, signal transducer and activator of transcription 1; Tneg, triple-negative.

All possible associations between the HRneg/Tneg index and the eight other signatures were explored in both the training (*n *= 199) and validation (*n *= 75) cohorts (Figure 3). Signature correlations (Pearson correlation coefficient, or R_p_) found to be significant and consistent between these two cohorts included the following: (a) positive associations between HRneg/Tneg and three different immune-related signatures (STAT1, IFN, and IR-7) and (b) positive associations among the five different proliferation module-containing signatures (MS-14, ONCO-RS, GGI, CSR/wound, and NKI-70). Consistent negative associations between the indices of the immune-related signatures and proliferation module-containing signatures were observed; however, some of these correlations did not reach significance.

To compare the relationships between the HRneg/Tneg and IR-7 prognostic indices with the degree of immune cell infiltration within the training and validation tumor cohorts, average expressions of T cell-specific and B cell-specific gene signatures were computed for each cohort. Since all of the genes in these lymphocyte-specific signatures are positively correlated with lymphocyte abundance, higher lymphocyte gene signature values within the cohorts represent higher degrees of lymphocytic infiltration. Owing to sign adjustments in the calculation of the HRneg/Tneg and IR-7 prognostic indices, negative correlations were expected to reflect the extent to which these indices were derived from infiltrating T or B lymphocytes. As shown in Table 3, modest, but significant, negative correlations were seen between the HRneg/Tneg index and both T-cell and B-cell gene expressions in the training and validation cohorts. More notably, however, the IR-7 signature correlated much more strongly with lymphocyte-specific gene expression, suggesting that it better reflects the extent of tumor infiltration by T cells and B cells whereas the HRneg/Tneg signature likely reflects additional non-lymphocytic tumor characteristics. Using the more restricted lymphocytic gene signatures containing only T- and B-cell surface markers resulted in very similar correlations with the two prognostic indices.

**Table 3:** Correlations (R_p_) between the HRneg/Tneg and IR-7 prognostic indices with T- and B-lymphocyte gene signatures in the training and validation tumor cohorts

	Training cohort (*n *= 199)				Validation cohort (*n *= 75)			
		
	HRneg/Tneg		IR-7		HRneg/Tneg		IR-7	
				
Lymphocyte-specific signatures	R_p_	*P *value	R_p_	*P *value	R_p_	*P *value	R_p_	*P *value
T-cell signature	-0.31	1.18 × 10^-5^	-0.65	< 2.2 × 10^-16^	-0.41	2.83 × 10^-4^	-0.62	3.439 × 10^-9^
T-cell co-receptor components	-0.39	9.28 × 10^-9^	-0.73	< 2.2 × 10^-16^	-0.42	1.78 × 10^-4^	-0.66	7.888 × 10^-11^
B-cell signature	-0.33	2.74 × 10^-6^	-0.89	< 2.2 × 10^-16^	-0.43	1.20 × 10^-4^	-0.77	9.07 × 10^-16^
B-cell surface co-receptor/marker	-0.15	2.94 × 10^-2^	-0.63	< 2.2 × 10^-16^	-0.34	2.60 × 10^-3^	-0.79	< 2.2 × 10^-16^

HRneg, hormone receptor-negative; IR-7, immune response signature with 7-gene immune response module; R_p_, Pearson correlation coefficient; Tneg, triple-negative.

### Pathway analysis of HRneg/Tneg index genes

**Figure 4:**
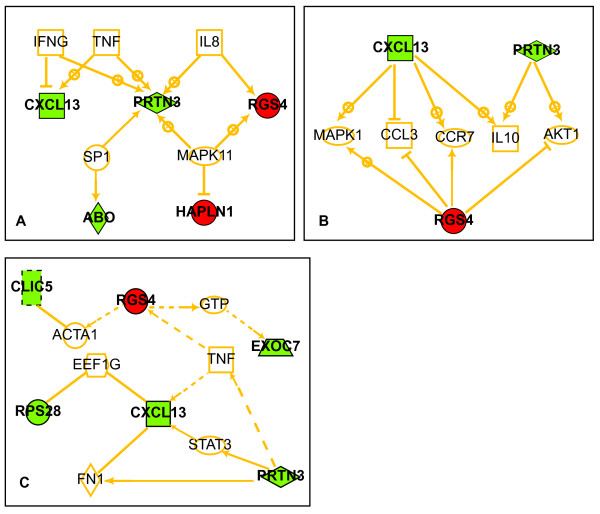
**Functional network connections between HRneg/Tneg signature genes**. **(a) **Pathway diagram linking HRneg/Tneg genes with common upstream regulators. **(b) **Pathway diagram linking HRneg/Tneg genes with common downstream effectors. **(c) **Pathway diagram linking HRneg/Tneg genes by their shortest path. (a-c) Genes associated with positive hazard ratios are red, and those associated with negative hazard ratios are green. Arrows with + denote upregulation, and those with ┴ denote inhibition. Solid lines signify direct gene-gene interactions, and broken lines represent relationships that may require secondary effectors not depicted in the network. HRneg, hormone receptor-negative; Tneg, triple-negative.

Ariadne Pathway Studio analysis identified well-known mediators of immune/inflammatory function (TNF, IL-8, and IFN-γ) and the proinflammatory cytokine/stress-activated kinase MAPK11 as potential common regulators of 4 of the 14 HRneg/Tneg index genes (Figure 4a). In addition, common downstream target analysis placed 3 of these HRneg/Tneg genes (CXCL13, RGS4, and PRTN3) as upstream of immune-function mediators IL-10, CCR7, and CCL3 (Figure 4b). Additional pathway exploration conducted using Ingenuity Pathway Systems (Figure 4c) identified cytokine TNF as linked to 6 HRneg/Tneg genes within a network that includes transcription factor STAT3, a key mediator of acute-phase response. Altogether, these network analyses identified 8 of the 14 genes within the HRneg/Tneg index as being potentially linked to immune/inflammatory cytokine regulation.

## Discussion

Our training (199 HRneg with 154 Tneg) and validation (75 HRneg with 46 Tneg) cohorts of node-negative, adjuvant treatment-naïve breast cancers showed distant metastatic event rates similar to that of another conservatively managed early-stage Tneg cohort [13]. Among the training set, 65 (33%) had an eventual metastatic relapse and 85% of these occurred within 5 years of diagnosis; among the validation set, 24 (32%) had a metastatic event, and 91% of these occurred within 5 years of diagnosis. Given this clinical behavior and the 77% preponderance of Tneg primary tumors in the training set, it may not be surprising that of the 11 top prognostic candidates entrained by the full HRneg cohort, four genes (CXCL13, CLIC5, RPS28, and MATN1) were also among the 7 top prognostic candidates independently entrained by the 154 Tneg tumors. The slightly different prognostic performances of individual genes within each training set are illustrated by CLIC5, MATN1, and RPS28, which were slightly more effective discriminators against the Tneg subset relative to the full set of HRneg tumors (Figures S1 and S2 in Additional files 3 and 4). It is interesting to note that higher expression of 12 of the 14 HRneg/Tneg genes is associated with better DMFS. This is consistent with observations among other HRneg outcome predictors (IR-7, STAT1, and IFN signatures) in which individual gene components are often more highly expressed in association with better prognosis [23-26], and this is in stark contrast with HRpos outcome predictors, in which elevated expression of the majority of gene components is associated with increased tumor proliferation and poor prognosis [22,23]. These inherent differences were taken into account during the computation of a composite signature index, such that a higher index value would be expected to correlate with worse DMFS outcome.

Relative to their gene-specific prognostic values, a composite signature score (index) based on all of the candidate genes proved to be a better discriminator of metastatic outcome. Despite their variable Cox coefficients, no attempt was made to individually weight each gene in generating the index. While indices computed from the HRneg and Tneg genes alone were able to dichotomize their respective training cohorts into groups with significant differences in DMFS (Figure S3 in Additional file 5), a combined index comprising all 14 HRneg/Tneg genes was able to achieve equivalent or better curve separation at both cut-points tested, thus providing a rationale for considering all 14 HRneg/Tneg genes together as a signature in further studies. Stepwise variable addition and removal analysis identified candidate subsets from which indices can be computed without loss of prognostic performance as assessed by univariate Cox analysis, suggesting that the HRneg/Tneg signature index has prognostic robustness. In particular, when the training cohort was dichotomized at a median value cut-point, the removal of up to three genes (for example, SSX3, MATN1, and PRTN3) did not significantly alter the HRs between poor versus good prognosis groups calculated from the modified 11-gene index relative to the full 14-gene index (HR 3.6, 95% CI 2.07 to 6.26 and HR 4.23, 95% CI 2.4 to 7.45, respectively). Also, a truncated index consisting of only 7 selected genes (CXCL13, CLIC5, RGS4, RPS28, RFX7, EXOC7, and HRBL) minimally altered the Kaplan-Meier curves and did not significantly reduce the HR (HR 3.6, 95% CI 2.09 to 6.23). Since both training and validation cohorts were composed of cases clinically annotated as HRneg, we reassessed the prognostic performance of the HRneg/Tneg index after removing 35 HRneg cases from the training cohort and 11 cases from the validation cohort having potentially high enough estrogen receptor transcript levels to be considered potentially false HRneg annotations. After these adjustments were made, the prognostic value of the HRneg/Tneg index improved slightly, as seen in the adjusted HRs calculated for the median cut-point dichotomized training and validation groups (HR 4.57, 95% CI 2.45 to 8.54 and HR 2.72, 95% CI 1.11 to 6.67, respectively).

Choice of cut-points may significantly influence a signature's prognostic performance. Thus, although significant curve separation was achieved in the full training data (and the Tneg subset) using the median index value as a cut-point, additional Kaplan-Meier analyses were conducted to identify an optimal cut-point that minimizes the log-rank *P *value. Care was taken to restrict these analyses to cut-points within the 20th and 80th percentiles to prevent extreme group sizes and reduce the likelihood of over-fitting the training data. Optimal curve separations were achieved at cut-points near the upper third quartile for the HRneg and Tneg indices in their respective training cohorts as well as the 14-gene HRneg/Tneg index in the full training data and the Tneg subset, suggesting that selecting an upper third quartile cut-point in future studies may yield optimal signature performance. This observation was independently confirmed in the validation cohort whose gene expression data were derived from two different expression microarray platforms; here, optimal curve separation was observed at an index value of 0.2087 and very close to the upper third quartile (0.2388). Interestingly, despite placing 75% of patients in the good-prognosis group, when accuracy was assessed as a function of the proportion of metastatic events based on high or low index values, the HRneg/Tneg index appeared highly accurate at identifying patients with good prognosis and less accurate at assigning patients to the poor-prognosis category, consistent with previous observations showing better negative predictive value and less-optimal positive predictive value for the IR gene signature [26]. These performance characteristics suggest that the HRneg/Tneg index may be well suited for identifying newly diagnosed, early-stage patients whose expected good outcome on conservative management would not mandate aggressive adjuvant chemotherapy. To explore this possibility further, we considered the distribution of HRneg/Tneg indices within the training and validation cohorts for those with either metastatic or non-metastatic outcomes (Figure S4 in Additional file 6); these distributions indicate that 10% to 15% of these cohorts have tumors with very low HRneg/Tneg indices and less than 10% likelihood of metastatic recurrence. However, additional independent validation studies are needed to confirm the presence of this small subgroup, which was not detected by the current optimization protocol and its survival characteristics.

The prognostic performance of the HRneg/Tneg index was also compared with that of other well-validated multigene predictors (Figure 2); in addition, the multiple predictive indices were compared with one another across both the training and validation cohorts (Figure 3). Non-optimized median values were used as cut-points for prognostic comparison purposes in the validation dataset to minimize bias toward the HRneg/Tneg signature. Despite these measures, only the HRneg/Tneg index demonstrated significant Kaplan-Meier curve separation, whereas the previously reported IR signature and two other immune-related signatures approached significance in our pooled validation cohort only. This is in contrast to signatures like ONCO-RS and MS-14 that were originally developed as HRpos outcome predictors and showed no prognostic value within this HRneg cohort and is in agreement with previous reports suggesting that prognoses of HRneg and HRpos breast cancers are driven by fundamentally different mechanisms [22,23]. While the strong correlations between different proliferation module-containing signatures were expected and in keeping with previous reports (as were the significant associations between the different immune-related signatures), the anti-correlations observed between the composite scores (index) of proliferation and immune signatures in HRneg breast cancers were not previously noted. It is worth noting that, owing to the adjustments made during index computations, these anti-correlations reflect a positive association between immune and proliferation function and may be in keeping with the growth-stimulatory effects of proinflammatory cytokine/chemokine signaling. These anti-correlations may also be attributed in part to the poor prognostic performances of proliferation module-containing signatures in HRneg breast cancers and may account for the lack of prognostic value of the HRneg/Tneg and IR signatures within a corresponding cohort of more than 400 node-negative, adjuvant-naïve HRpos breast cancers ([GEO:GSE2034, GSE7390], NKI-295) in which both the ONCO-RS and MS-14 signatures are significantly prognostic (data not shown).

Given these signature associations, it is not entirely surprising that network analysis of the HRneg/Tneg signature employing two different commercial pathway programs revealed no links to known proliferation pathways but showed direct and indirect connections to several immune/inflammatory nodes, with 8 of the 14 HRneg/Tneg signature genes functionally linked to chemokine regulation and expression (Figure 4). Although the IR, IFN, and STAT1 signature genes are not components of the HRneg/Tneg signature, one gene in this index, CXCL13, was found to correlate significantly with each of the 7 IR genes, suggesting surrogate representation of the IR-7 index within the HRneg/Tneg signature and probably accounting for the weak but consistent association observed between the HRneg/Tneg index and the three other immune-related signatures in both training and validation cohorts. The observation that these three other immune-related signatures correlated much more strongly among themselves supports the possibility that the 14-gene HRneg/Tneg signature contains other non-immune/inflammatory modules, although examples of such pathways were not apparent in our network analysis. To pursue this hypothesis further, we attempted to correlate both the HRneg/Tneg and IR-7 indices with an assessment of lymphocyte infiltration within the cohort tumors. Only a small subset of the dataset tumors was clinically annotated for degree of lymphocytic infiltration [6,29], and an initial analysis of these few Tneg cases suggested a possible trend between the HRneg/Tneg score and the degree of lymphocytic infiltration (data not shown). Therefore, using a reported set of T cell- and B cell-specific genes as surrogate signatures for lymphocytic infiltration in the training and validation cases (Table S2 in Additional file 2) [34], we demonstrated a modest correlation between the HRneg/Tneg index and both T-cell and B-cell gene expression. By comparison (Table 3), the IR-7 index correlated much more strongly with these lymphocyte-specific gene expression signatures, indicating that the IR-7 index may largely represent the extent of tumor infiltration by T cells and B cells and the HRneg/Tneg index potentially reflects this as well as additional tumor epithelial characteristics.

Of the chemokine-associated genes in the HRneg/Tneg index, CXCL13 (ligand for the chemokine receptor CXCR5) has been best studied in breast cancer and recently was shown to be the most significantly overexpressed (mRNA and protein) chemokine in a panel of early-stage human breast cancers following a survey of 84 different chemokines [35]. Surprisingly, in this study, breast cancer overexpression of CXCL13 did not correlate with tumor infiltration by leukocytes but instead was immunohistochemically localized to the cytoplasm of the malignant epithelial cells [35]. This study also illustrates the possibility that some HRneg/Tneg signature genes emerge as blood biomarkers since CXCL13 blood levels were found to be specifically increased in patients with breast cancer [35]. Another chemokine-associated HRneg/Tneg gene initially thought to be expressed only in activated neutrophils, PRTN3 (neutrophil-derived serine proteinase 3), was recently shown to be transcriptionally overexpressed in cytokine-exposed epithelial cells, although its expression has not yet been linked to cancer [36,37]. Other chemokine-associated genes in the HRneg/Tneg signature linked to both epithelial and cancer cell expression include EXOC7 (exocyst complex component 7) [38], ABO (blood group glycosylransferases A and B) [39], CLIC5 (chloride intracellular channel 5) [40], RPS28 (40S ribosomal protein S28) [41], HAPLN1 (hyaluronan- and proteoglycan-linked protein 1) [42], and RGS4 (regulator of G-protein signaling 4) [43-46]. Studies of the last gene also illustrate why transcriptome-derived cancer signatures cannot reliably be extrapolated to protein-based tumorigenic mechanisms without more in-depth evaluation. While RGS4 transcriptional upregulation has been associated with increased viability, invasion, and motility of thyroid cancer, glioma, ovarian ascites, and Tneg breast cancer cells, it has now been shown that RGS4 mRNA and protein levels do not correlate, since (despite high RGS4 transcript levels) RGS4 protein levels must be proteasomally downregulated to enable metastasis [46]. To date, none of the 6 other HRneg/Tneg signature genes not functionally linked to chemokine pathways (PRRG3, RFX7, MATN1, SSX3, HRBL, and ZNF3) has shown any reported association with cancer.

## Conclusions

A 14-gene HRneg/Tneg prognostic signature was identified from pooled expression microarray data from HRneg and Tneg breast cancer cases (node-negative, adjuvant-naïve) assigned for signature training (*n *= 199 cases) and validation (*n *= 75 cases). In both pooled cohorts, the HRneg/Tneg summation index proved prognostically superior to a recently described IR-7 gene signature derived from different HRneg training and validation cases, although expression of one gene in the HRneg/Tneg signature (CXCL13) appears to correlate with all components in the IR-7 signature, which, in turn, correlates strongly with other reported immune-related gene signatures and the extent of tumor infiltration by lymphocytes. In contrast, previously described multigene predictors known to contain proliferation modules are shown to have no prognostic value in these HRneg and Tneg breast cancer cohorts. Over half of the genes in the HRneg/Tneg prognostic signature show network and pathway links to chemokine expression; however, the HRneg/Tneg index may reflect both immune cell infiltration as well as tumor epithelial characteristics since many of the signature-associated chemokines are known to be expressed by epithelial cells. Further validation of this HRneg/Tneg prognostic signature is now in progress following transfer to a different assay platform (reverse transcription-polymerase chain reaction) suitable for use on archived and clinical samples of formalin-fixed and paraffin-embedded breast cancers.

## Abbreviations

CI: confidence interval; CSR: core serum response; DMFS: distant metastasis-free survival; DWD: distance-weighted discrimination; GGI: genomic grade index; HER2/ERBB2: human epidermal growth factor receptor 2; HR: hazard ratio; HRneg: hormone receptor-negative; HRpos: hormone receptor-positive; IFN: interferon; IR: immune response; IR-7: immune response signature with 7-gene immune response module; MS-14: Celera 14-gene metastasis score; NKI-70: 70-gene Mammaprint signature; ONCO-RS: Oncotype/Genomic Health recurrence score; PAM: prediction analysis of microarrays; STAT1: signal transducer and activator of transcription 1; Tneg: triple-negative.

## Competing interests

CY, LE, FW, and CCB are named as inventors of the above-mentioned HRneg/Tneg prognostic gene signature in a joint institutional patent application filed by the University of California, San Francisco and the Buck Institute for Age Research. No financial or other support of any kind has resulted from this patent application. The other authors declare that they have no competing interests.

## Authors' contributions

CY identified all of the public datasets, carried out all of the biostatistical and informatic analyses, and helped draft the manuscript. LE co-initiated the project, helped guide the study design, and participated in formulating the study conclusions. DHM supervised and participated in the biostatistical analyses. FW and JS participated in the study design, guided the informatic analyses, and helped formulate the study conclusions. CCB conceived and coordinated the project, supervised all data curation and analysis, formulated the study conclusions, and drafted the final manuscript. All authors read and approved the final manuscript.

## Supplementary Material

Additional file 1 **Supplemental table S1**. Summary of patient characteristics (grade, tumor size and number of samples scored for lymphocytic infiltration) by data source. "na" denotes where this annotation is not available to the public; and "nd" represents cohorts where Tneg status by ERBB2 transcript levels were not determined. Click here for file

Additional file 2 **Supplemental table S2**. Established multigene signatures assessed in comparison to HRneg/Tneg signatures. Signatures annotated for Affymetrix probe set information (STAT1 and GGI) are mapped to training data using the Affymetrix probe set ID; otherwise, signatures are mapped using gene symbols. Only signature components that can be mapped (as denoted by a "Y" in the "Mapped to Training Set" or "Mapped to Validation Set" columns) are included in the computation of signature indices in accordance to their reported correlation with prognosis (as denoted in the "Contribution to Index" column). Click here for file

Additional file 3 **Supplemental figure S1**. Prognostic performance of individual HRneg genes in training cohort. Kaplan-Meier plots of distant metastatic events dichotomized at the median by high (red) or low (green) expression of individual HRneg genes in training cohort of 199 HRneg cases. Significant differences in survival between groups were determined by log rank analysis. Click here for file

Additional file 4 **Supplemental figure S2**. Prognostic performance of individual Tneg genes in training cohort. Kaplan-Meier plots of distant metastatic events dichotomized at the median by high (red) or low (green) expression of individual Tneg genes in training cohort subset of 154 Tneg cases. Significant differences in survival between groups were determined by log rank analysis. Click here for file

Additional file 5 **Supplemental figure S3**. Prognostic performance of the 11-gene HRneg and 7-gene Tneg indices considered independently. Kaplan-Meier plots of distant-metastatic events dichotomized at the upper 3^rd ^quartile by high (red) or low (green) expression indices of (A) the 11 prognostic gene candidates identified from the 199 HRneg training cases; and (B) the 7 prognostic gene candidates identified from the subset of 154 Tneg training cases. Click here for file

Additional file 6 **Supplemental figure S4**. Distribution of HRneg/Tneg scores by cohort and outcome. The histograms of HRneg/Tneg scores among cases with metastatic (red) or non-metastatic (blue) outcome within the (A) training and (B) validation cohorts. Red dotted-line boxes labeled "worst prognosis group" highlight cases within the upper 3^rd ^quartile of HRneg/Tneg scores, corresponding to the "High" index groups shown in Figures 1A and 1C. Green dotted-line boxes labeled 'best prognosis group' highlight cases with very low index values (lowest ~15% in training, and ~11% in validation cohorts) with better than 90% DMFS. Click here for file

## References

[B1] Anders C Carey LA Understanding and treating triple-negative breast cancer Oncology 2008 22 1233 1243 18980022 PMC2868264

[B2] Voduc D Nielsen T Basal and triple-negative breast cancers: impact on clinical decision-making and novel therapeutic options Clin Breast Cancer 2008 8 s171 s178 10.3816/CBC.2008.s.014 19158038

[B3] Rakha EA Ellis IO Triple-negative/basal-like breast cancer: review Pathology 2009 41 40 47 10.1080/00313020802563510 19089739

[B4] Chen XS Ma CD Wu JY Yang WT Lu H Wu J Lu JS Shao ZM Shen ZZ Shen KW Molecular subtype approximated by quantitative estrogen receptor, progesterone receptor and Her2 can predict the prognosis of breast cancer Tumori 2010 96 103 110 20437866 10.1177/030089161009600117

[B5] Sorlie T Perou CM Tibshirani R Aas T Geisler S Johnsen H Hastie T Eisen MB van de Rijn M Jeffrey SS Thorsen T Quist H Matese JC Brown PO Botstein D Lonning PE Borresen-Dale AL Gene expression patterns of breast carcinomas distinguish tumor subclasses with clinical implications Proc Natl Acad Sci USA 2001 98 10869 10874 10.1073/pnas.191367098 11553815 PMC58566

[B6] van't Veer LJ Dai H van de Vijver MJ He YD Hart AAM Mao M Peterse HL van der Kooy K Marton MJ Witteveen AT Schreiber GJ Kerkhoven RM Roberts C Linsley PS Bernards R Friend SH Gene expression profiling predicts clinical outcome of breast cancer Nature 2002 415 530 536 10.1038/415530a 11823860

[B7] Chin K DeVries S Fridlyand J Spellman PT Roydasgupta R Kuo WL Lapuk A Neve RM Qian Z Ryder T Chen F Feiler H Tokuyasu T Kingsley C Dairkee S Meng Z Chew K Pinkel D Jain A Ljung BM Esserman L Albertson DG Waldman FM Gray JW Genomic and transcriptional aberrations linked to breast cancer pathophysiologies Cancer Cell 2006 10 529 541 10.1016/j.ccr.2006.10.009 17157792

[B8] Ross JS Hatzis C Symmans WF Pusztai L Hortobagyi GN Commercialized multigene predictors of clinical outcome for breast cancer Oncologist 2008 13 477 493 10.1634/theoncologist.2007-0248 18515733

[B9] Pusztai L Gene expression profiling of breast cancer Breast Cancer Res 2009 11 S11 10.1186/bcr2430 20030862 PMC2797691

[B10] Voduc KD Cheang MCU Tyldesley S Gelmon K Nielsen TO Kennecke H Breast cancer subtypes and the risk of local and regional relapse J Clin Oncol 2010 28 1684 1691 10.1200/JCO.2009.24.9284 20194857

[B11] Kassam F Enright K Dent R Dranitsaris G Myers J Flynn C Fralick M Kumar R Clemons M Survival outcomes for patients with metastatic triple-negative breast cancer: implications for clinical practice and trial design Clin Breast Cancer 2009 9 29 33 10.3816/CBC.2009.n.005 19299237

[B12] Dent R Trudeau M Pritchard KI Hanna WM Kahn HK Sawka CA Lickley LA Rawlinson E Sun P Narod SA Triple-negative breast cancer: clinical features and patterns of recurrence Clin Cancer Res 2007 13 4429 4434 10.1158/1078-0432.CCR-06-3045 17671126

[B13] Haffty BG Yang Q Reiss M Kearney T Higgins SA Weidhaas J Harris L Hait W Toppmeyer D Locoregional relapse and distant metastasis in conservatively managed triple negative early-stage breast cancer J Clin Oncol 2006 24 5652 5657 10.1200/JCO.2006.06.5664 17116942

[B14] Jemal A Siegel R Ward E Hao Y Xu J Thun MJ Cancer Statistics, 2009 CA Cancer J Clin 2009 59 225 249 10.3322/caac.20006 19474385

[B15] Cheang MCU Chia SK Voduc D Gao D Leung S Snider J Watson M Davies S Bernard PS Parker JS Perou CM Ellis MJ Nielsen TO Ki67 index, HER2 status, and prognosis of patients with luminal B breast cancer J Natl Cancer Inst 2009 101 736 750 10.1093/jnci/djp082 19436038 PMC2684553

[B16] Tutt A Wang A Rowland C Gillett C Lau K Chew K Dai H Kwok S Ryder K Shu H Springall R Cane P McCallie B Kam-Morgan L Anderson S Buerger H Gray J Bennington J Esserman L Hastie T Broder S Sninsky J Brandt B Waldman F Risk estimation of distant metastasis in node-negative, estrogen receptor-positive breast cancer patients using an RT-PCR based prognostic expression signature BMC Cancer 2008 8 339 10.1186/1471-2407-8-339 19025599 PMC2631011

[B17] Wang Y Klijn JGM Zhang Y Sieuwerts AM Look MP Yang F Talantov D Timmermans M Meijer-van Gelder ME Yu J Jatkoe T Berns EMJJ Atkins D Foekens JA Gene-expression profiles to predict distant metastasis of lymph-node-negative primary breast cancer Lancet 2005 365 671 679 15721472 10.1016/S0140-6736(05)17947-1

[B18] Chang HY Nuyten DSA Sneddon JB Hastie T Tibshirani R Sørlie T Dai H He YD van't Veer LJ Bartelink H van de Rijn M Brown PO van de Vijver MJ Robustness, scalability, and integration of a wound-response gene expression signature in predicting breast cancer survival Proc Natl Acad Sci USA 2005 102 3738 3743 10.1073/pnas.0409462102 15701700 PMC548329

[B19] Paik S Shak S Tang G Kim C Baker J Cronin M Baehner FL Walker MG Watson D Park T Hiller W Fisher ER Wickerham DL Bryant J Wolmark N A multigene assay to predict recurrence of tamoxifen-treated, node-negative breast cancer N Engl J Med 2004 351 2817 2826 10.1056/NEJMoa041588 15591335

[B20] Miller LD Smeds J George J Vega VB Vergara L Ploner A Pawitan Y Hall P Klaar S Liu ET Bergh J An expression signature for p53 status in human breast cancer predicts mutation status, transcriptional effects, and patient survival Proc Natl Acad Sci USA 2005 102 13550 13555 10.1073/pnas.0506230102 16141321 PMC1197273

[B21] Sotiriou C Wirapati P Loi S Harris A Fox S Smeds J Nordgren H Farmer P Praz V Haibe-Kains B Desmedt C Larsimont D Cardoso F Peterse H Nuyten D Buyse M Van de Vijver MJ Bergh J Piccart M Delorenzi M Gene expression profiling in breast cancer: understanding the molecular basis of histologic grade to improve prognosis J Natl Cancer Inst 2006 98 262 272 10.1093/jnci/djj052 16478745

[B22] Wirapati P Sotiriou C Kunkel S Farmer P Pradervand S Haibe-Kains B Desmedt C Ignatiadis M Sengstag T Schutz F Goldstein D Piccart M Delorenzi M Meta-analysis of gene expression profiles in breast cancer: toward a unified understanding of breast cancer subtyping and prognosis signatures Breast Cancer Res 2008 10 R65 10.1186/bcr2124 18662380 PMC2575538

[B23] Desmedt C Haibe-Kains B Wirapati P Buyse M Larsimont D Bontempi G Delorenzi M Piccart M Sotiriou C Biological processes associated with breast cancer clinical outcome depend on the molecular subtypes Clin Cancer Res 2008 14 5158 5165 10.1158/1078-0432.CCR-07-4756 18698033

[B24] Hu Z Fan C Oh DS Marron JS He X Qaqish BF Livasy C Carey LA Reynolds E Dressler L Nobel A Parker J Ewend MG Sawyer LR Wu J Liu Y Nanda R Tretiakova M Ruiz Orrico A Dreher D Palazzo JP Perreard L Nelson E Mone M Hansen H Mullins M Quackenbush JF Ellis MJ Olopade OI Bernard PS Perou CM The molecular portraits of breast tumors are conserved across microarray platforms BMC Genomics 2006 7 96 10.1186/1471-2164-7-96 16643655 PMC1468408

[B25] Teschendorff A Miremadi A Pinder S Ellis I Caldas C An immune response gene expression module identifies a good prognosis subtype in estrogen receptor negative breast cancer Genome Biol 2007 8 R157 10.1186/gb-2007-8-8-r157 17683518 PMC2374988

[B26] Teschendorff A Caldas C A robust classifier of high predictive value to identify good prognosis patients in ER-negative breast cancer Breast Cancer Res 2008 10 R73 10.1186/bcr2138 18755024 PMC2575547

[B27] Kreike B van Kouwenhove M Horlings H Weigelt B Peterse H Bartelink H van de Vijver M Gene expression profiling and histopathological characterization of triple-negative/basal-like breast carcinomas Breast Cancer Res 2007 9 R65 10.1186/bcr1771 17910759 PMC2242660

[B28] Minn AJ Gupta GP Padua D Bos P Nguyen DX Nuyten D Kreike B Zhang Y Wang Y Ishwaran H Foekens JA van de Vijver M Massagué J Lung metastasis genes couple breast tumor size and metastatic spread Proc Natl Acad Sci USA 2007 104 6740 6745 10.1073/pnas.0701138104 17420468 PMC1871856

[B29] Desmedt C Piette F Loi S Wang Y Lallemand Fo Haibe-Kains B Viale G Delorenzi M Zhang Y d'Assignies MS Bergh J Lidereau R Ellis P Harris AL Klijn JGM Foekens JA Cardoso F Piccart MJ Buyse M Sotiriou C Strong time dependence of the 76-gene prognostic signature for node-negative breast cancer patients in the TRANSBIG multicenter independent validation series Clin Cancer Res 2007 13 3207 3214 10.1158/1078-0432.CCR-06-2765 17545524

[B30] Marron JS Todd MJ Ahn J Distance-weighted discrimination JASA 2007 102 1267 1271

[B31] Loi S Haibe-Kains B Desmedt C Lallemand F Tutt AM Gillet C Ellis P Harris A Bergh J Foekens JA Klijn JGM Larsimont D Buyse M Bontempi G Delorenzi M Piccart MJ Sotiriou C Definition of clinically distinct molecular subtypes in estrogen receptor-positive breast carcinomas through genomic grade J Clin Oncol 2007 25 1239 1246 10.1200/JCO.2006.07.1522 17401012

[B32] van de Vijver MJ He YD van 't Veer LJ Dai H Hart AAM Voskuil DW Schreiber GJ Peterse JL Roberts C Marton MJ Parrish M Atsma D Witteveen A Glas A Delahaye L van der Velde T Bartelink H Rodenhuis S Rutgers ET Friend SH Bernards R A gene-expression signature as a predictor of survival in breast cancer N Engl J Med 2002 347 1999 2009 10.1056/NEJMoa021967 12490681

[B33] Broad Institute homepage http://www.broadinstitute.org/

[B34] Palmer C Diehn M Alizadeh A Brown P Cell-type specific gene expression profiles of leukocytes in human peripheral blood BMC Genomics 2006 7 115 10.1186/1471-2164-7-115 16704732 PMC1479811

[B35] Panse J Friedrichs K Marx A Hildebrandt Y Luetkens T Bartels K Horn C Stahl T Cao Y Milde-Langosch K Niendorf A Kroger N Wenzel S Leuwer R Bokemeyer C Hegewisch-Becker S Atanackovic D Chemokine CXCL13 is overexpressed in the tumour tissue and in the peripheral blood of breast cancer patients Br J Cancer 2008 99 930 938 10.1038/sj.bjc.6604621 18781150 PMC2538749

[B36] Korkmaz B Moreau T Gauthier F Neutrophil elastase, proteinase 3 and cathepsin G: Physicochemical properties, activity and physiopathological functions Biochimie 2008 90 227 242 10.1016/j.biochi.2007.10.009 18021746

[B37] Uehara A Sugawara Y Sasano T Takada H Sugawara S Proinflammatory cytokines induce proteinase 3 as membrane-bound and secretory forms in human oral epithelial cells and antibodies to proteinase 3 activate the cells through protease-activated receptor-2 J Immunol 2004 173 4179 4189 15356169 10.4049/jimmunol.173.6.4179

[B38] Liu J Yue P Artym VV Mueller SC Guo W The role of the exocyst in matrix metalloproteinase secretion and actin dynamics during tumor cell invadopodia formation Mol Biol Cell 2009 20 3763 3771 10.1091/mbc.E08-09-0967 19535457 PMC2777935

[B39] Nakagoe Nakagoe T Fukushima Fukushima K Itoyanagi Itoyanagi N Ikuta Ikuta Y Oka Oka T Nagayasu Nagayasu T Ayabe Ayabe H Hara Hara S Ishikawa Ishikawa H Minami Minami H Expression of ABH/Lewis-related antigens as prognostic factors in patients with breast cancer J Cancer Res Clin Oncol 2002 128 257 264 10.1007/s00432-002-0334-5 12029441 PMC12164471

[B40] Furuta J Nobeyama Y Umebayashi Y Otsuka F Kikuchi K Ushijima T Silencing of peroxiredoxin 2 and aberrant methylation of 33 CpG islands in putative promoter regions in human malignant melanomas Cancer Res 2006 66 6080 6086 10.1158/0008-5472.CAN-06-0157 16778180

[B41] Otsuka M Kato M Yoshikawa T Chen H Brown EJ Masuho Y Omata M Seki N Differential expression of the L-plastin gene in human colorectal cancer progression and metastasis Biochem Biophys Res Commun 2001 289 876 881 10.1006/bbrc.2001.6047 11735128

[B42] Ivanova AV Goparaju CMV Ivanov SV Nonaka D Cruz C Beck A Lonardo F Wali A Pass HI Protumorigenic role of HAPLN1 and its IgV domain in malignant pleural mesothelioma Clin Cancer Res 2009 15 2602 2611 10.1158/1078-0432.CCR-08-2755 19351750 PMC3761224

[B43] Tatenhorst L Senner V Püttmann S Paulus W Regulators of G-protein signaling 3 and 4 (RGS3, RGS4) are associated with glioma cell motility J Neuropathol Exp Neurol 2004 63 210 222 15055445 10.1093/jnen/63.3.210

[B44] Puiffe ML Le Page C Filali-Mouhim A Zietarska M Ouellet V Tonin PN Chevrette M Provencher DM Mes-Masson AM Characterization of ovarian cancer ascites on cell invasion, proliferation, spheroid formation, and gene expression in an in vitro model of epithelial ovarian cancer Neoplasia 2007 9 820 829 10.1593/neo.07472 17971902 PMC2040209

[B45] Nikolova D Zembutsu H Sechanov T Vidinov K Kee L Ivanova R Becheva E Kocova M Toncheva D Nakamura Y Genome-wide gene expression profiles of thyroid carcinoma: identification of molecular targets for treatment of thyroid carcinoma Oncol Rep 2008 20 105 121 18575725

[B46] Xie Y Wolff DW Wei T Wang B Deng C Kirui JK Jiang H Qin J Abel PW Tu Y Breast cancer migration and invasion depend on proteasome degradation of regulator of G-protein signaling 4 Cancer Res 2009 69 5743 5751 10.1158/0008-5472.CAN-08-3564 19549919 PMC2741027

