# Supplementary figures and images for: A multigene predictor of metastatic outcome in early stage hormone receptor-negative and triple-negative breast cancer
Source: Breast Cancer Res. 2010 Oct 14;12(5):R85. doi: 10.1186/bcr2753 (PMC3096978; doi:10.1186/bcr2753)

204338\_s\_at (RGS4)

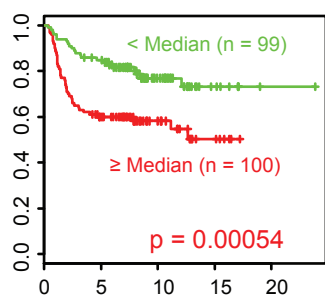

205242\_at (CXCL13)

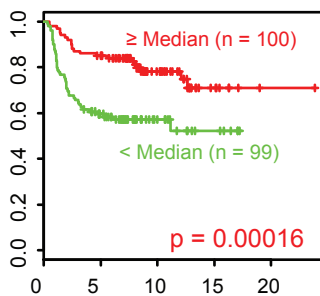

205523\_at (HAPLN1)

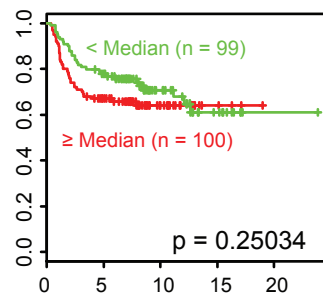

206904\_at (MATN1)

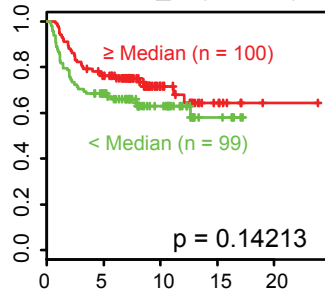

207341\_at (PRTN3)

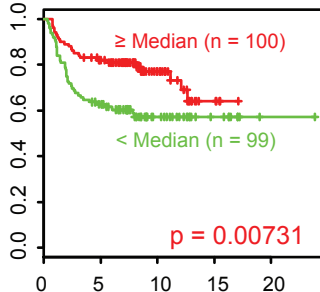208902\_s\_at  
(FLJ46061 /// RPS28)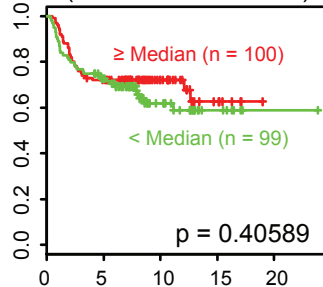

212035\_s\_at (EXOC7)

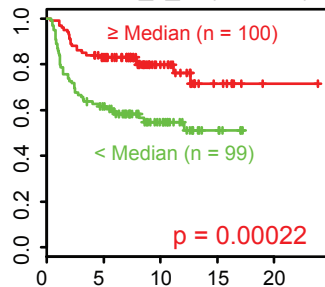

216929\_x\_at (ABO)

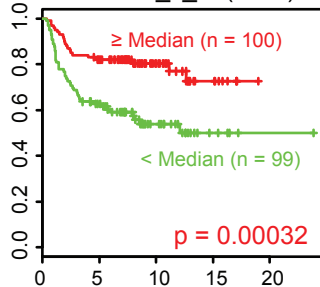

217628\_at (CLIC5)

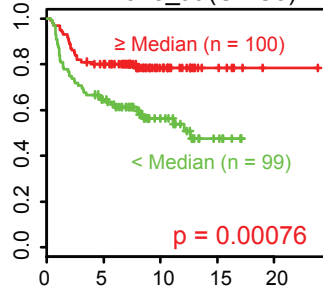

218430\_s\_at (RFXDC2)

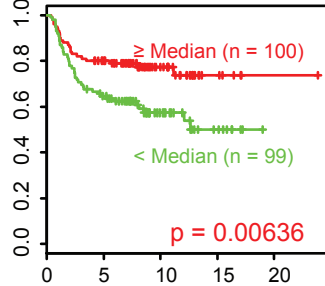

220433\_at (PRRG3)

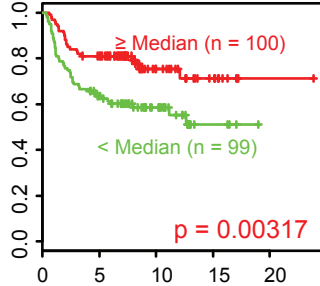

Supplement: Additional file 3 — Supplemental figure S1. Prognostic performance of individual HRneg genes in training cohort. Kaplan-Meier plots of distant metastatic events dichotomized at the median by high (red) or low (green) expression of individual HRneg genes in training cohort of 199 HRneg cases. Significant differences in survival between groups were determined by log rank analysis. [file bcr2753-S3.PDF]

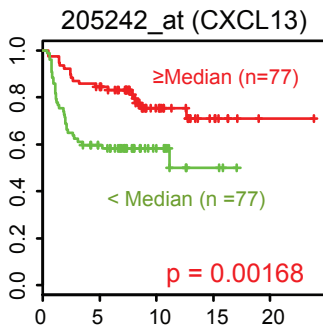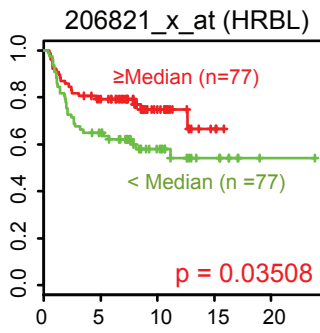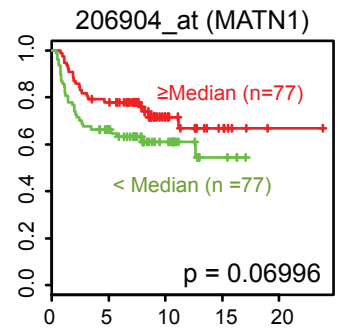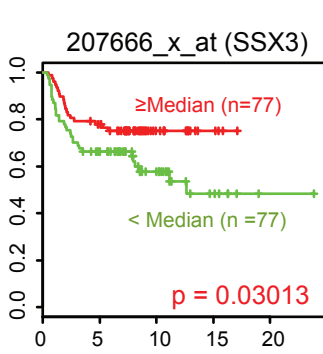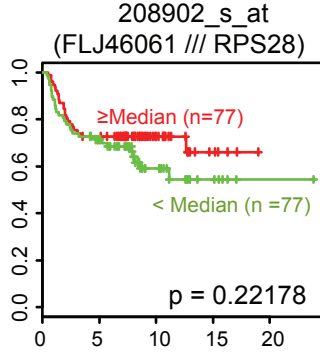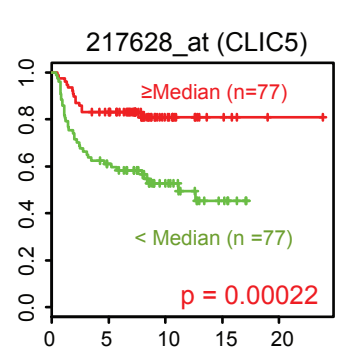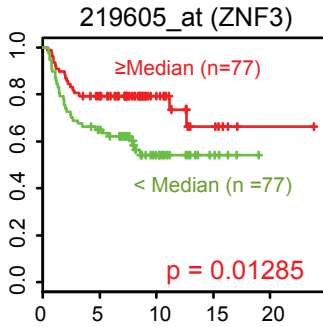

Supplement: Additional file 4 — Supplemental figure S2. Prognostic performance of individual Tneg genes in training cohort. Kaplan-Meier plots of distant metastatic events dichotomized at the median by high (red) or low (green) expression of individual Tneg genes in training cohort subset of 154 Tneg cases. Significant differences in survival between groups were determined by log rank analysis. [file bcr2753-S4.PDF]

**A**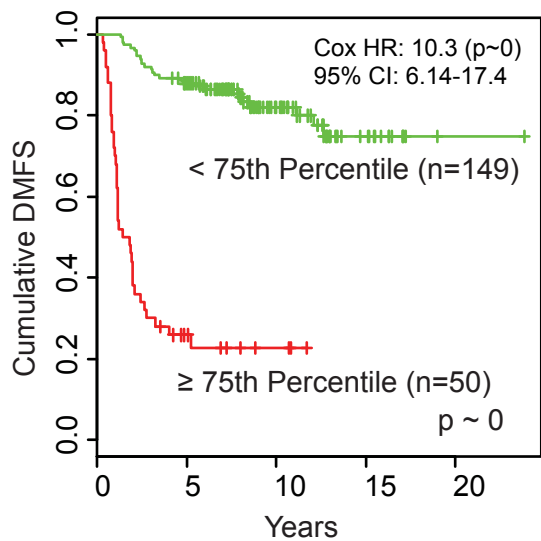**B**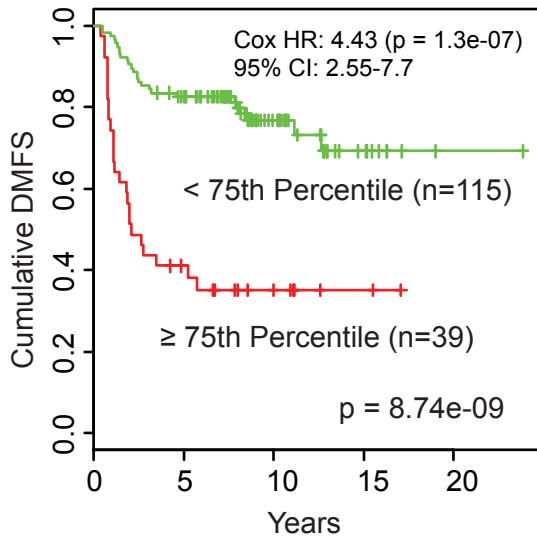

Supplement: Additional file 5 — Supplemental figure S3. Prognostic performance of the 11-gene HRneg and 7-gene Tneg indices considered independently. Kaplan-Meier plots of distant-metastatic events dichotomized at the upper 3rd quartile by high (red) or low (green) expression indices of (A) the 11 prognostic gene candidates identified from the 199 HRneg training cases; and (B) the 7 prognostic gene candidates identified from the subset of 154 Tneg training cases. [file bcr2753-S5.PDF]

A

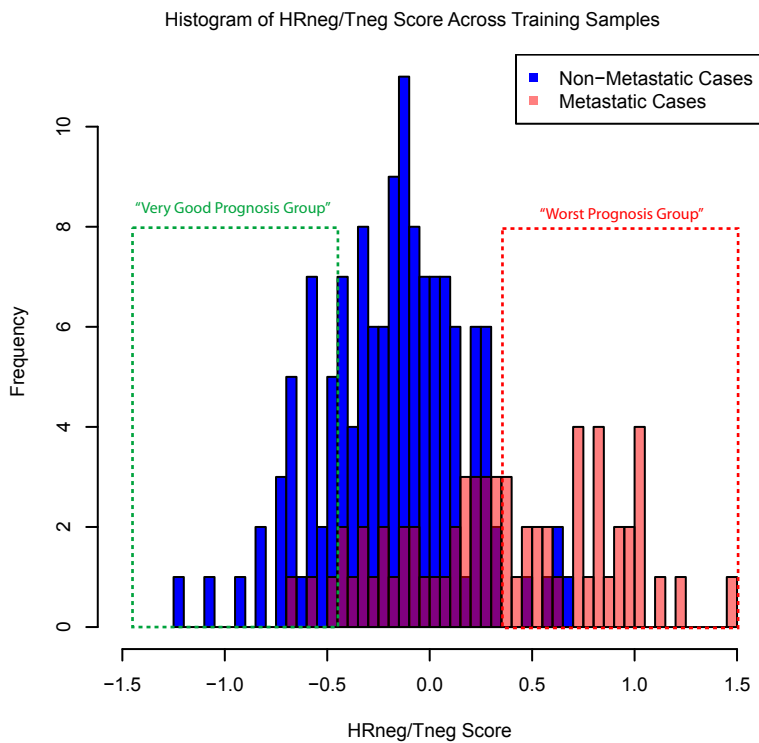

B

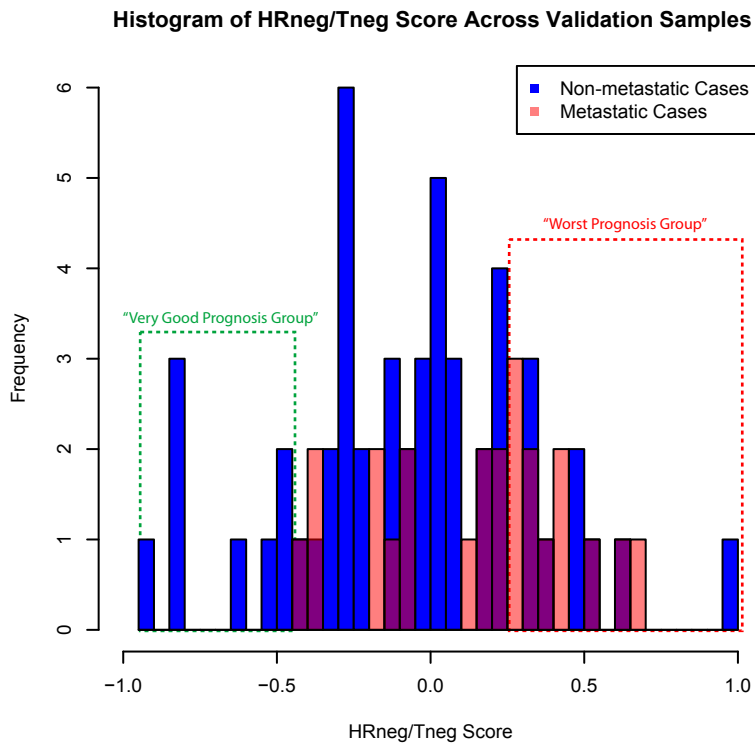

Supplement: Additional file 6 — Supplemental figure S4. Distribution of HRneg/Tneg scores by cohort and outcome. The histograms of HRneg/Tneg scores among cases with metastatic (red) or non-metastatic (blue) outcome within the (A) training and (B) validation cohorts. Red dotted-line boxes labeled "worst prognosis group" highlight cases within the upper 3rd quartile of HRneg/Tneg scores, corresponding to the "High" index groups shown in Figures 1A and 1C. Green dotted-line boxes labeled 'best prognosis group' highlight cases with very low index values (lowest ~15% in training, and ~11% in validation cohorts) with better than 90% DMFS. [file bcr2753-S6.PDF]
